# The R package for DICOM to brain imaging data structure conversion

**DOI:** 10.1038/s41597-023-02583-4

**Published:** 2023-10-04

**Authors:** Niklas Wulms, Sven Eppe, Mahboobeh Dehghan-Nayyeri, Adam J. Streeter, Nadine Bonberg, Klaus Berger, Benedikt Sundermann, Heike Minnerup

**Affiliations:** 1 Institute of Epidemiology and Social Medicine, University of Muenster, Muenster, Germany; 2 State Cancer Registry of North Rhine-Westphalia, Bochum, Germany; 3 Department of Psychosomatic Medicine and Psychotherapy, LVR Clinic, Medical Faculty of the Heinrich-Heine-University Duesseldorf, Duesseldorf, Germany; 4 Clinic of Radiology, University Hospital Muenster, Muenster, Germany; 5 Institute of Radiology and Neuroradiology, Evangelisches Krankenhaus, Medical Campus, University of Oldenburg, Oldenburg, Germany; 6 Research Center Neurosensory Science, University of Oldenburg, Oldenburg, Germany

**Keywords:** Software, Standards, Brain, Image processing

## Abstract

The BIDSconvertR package is the first R-based tool for organizing magnetic resonance imaging (MRI) research data in accordance with the Brain Imaging Data Structure (BIDS) specification. Key features are the DICOM (Digital Imaging and Communications in Medicine) to NIfTI (Neuroimaging Informatics Technology Initiative) and NIfTI to BIDS conversion, the implementation of the BIDS Validator and a MRI data viewer to efficiently manage MRI neuroimaging data sets. The BIDSconvertR offers an interactive user dialogue and a graphical user interface. BIDS validation is facilitated by color-coding of the BIDS sequence-IDs. Data cleaning is simplified by the option of using regular expressions. The BIDSconvertR contributes to the growing efforts to improve reproducibility in neuroimaging research by facilitating researchers to share and organize data in a standardized and transparent manner.

## Introduction

When it comes to effective research data management, there are various challenges to meet, including different data and folder structures, as well as naming conventions. The need for distinct specifications to prevent untidy data becomes increasingly important as data sets grow larger and more complex. Neuroimaging data and metadata, for instance, are named and organized in a variety of ways across researchers and laboratories. As a result, data analysis pipelines (preprocessing or statistical) that have been developed for a specific data set can not simply be applied to other data sets, which increases efforts and impairs reproducibility. The DICOM format was a significant milestone in the standardization of medical neuroimage storage and communication. However, it is not human-readable and typically consists of many files per image. As a more workable option for scientific use the NIfTI standard was created. It can be directly converted from MRI DICOM data, has a human-readable format and consists of one file per image^1^. More recently, the Brain Imaging Data Structure (BIDS) was created by the BIDS-consortium as a standardized method of storing and reporting neuroimaging data (MRI, MEG, and EEG) alongside corresponding behavioral or clinical data^2^. The purpose of this specification is to facilitate research collaborations, methodological developments (such as pre-processing and analysis pipelines) and replication studies in the field of neuroimaging^3^. There are multiple tools (https://bids.neuroimaging.io/benefits.html) that can convert DICOM to NIfTI (such as dcm2niix^4^ that is used by our tool), NIfTI to BIDS, or both. However, advanced programming knowledge in Python or Matlab is often required to use the BIDS conversion software. Only recently, the first few tools have been released that present a user-friendly graphical user interface^5^.

Here, we introduce the ‘BIDSconvertR’^6^, the first package for BIDS conversion developed in R. It covers every step from DICOM input to BIDS output. The tool is suitable for users with less programming experience as it mainly works with pop-up boxes and graphical user interfaces. It converts DICOM to NIfTI with the widely distributed and validated dcm2niix^4^. The usability is optimized by an interactive user dialogue to select the input and output folder, the option to define the subject-ID or remove patterns with regular expressions, the color-coded validation of BIDS sequence-IDs and validation of the resulting data set with the BIDS-Validator^7^. It also features ‘Shiny BIDS’, an interactive MRI viewer based on papaya that allows users to effortlessly browse through the layered BIDS folder structure and to manually assess image quality.

This publication aims to introduce the tool to the scientific community by detailing the prerequisites, usage, functionality, and features of the ‘BIDSconvertR’^6^ (https://github.com/bidsconvertr/bidsconvertr). It is accompanied by a detailed documentation website (https://bidsconvertr.github.io/) and an online tutorial (https://bidsconvertr.github.io/tutorial/).

## Results

BIDSconvertR was initially developed and validated on MRI data from the BiDirect study^8,9^, a data set with more than 90,000 single images (for details of the study, see *Methods*). It was used to convert the DICOM data of all participants to a valid BIDS^2^ data set. The performance of BIDSconvertR was also tested by the coauthors on different operating systems (Ubuntu 22.04 and Windows 10), different MRI vendors (Siemens and Philips) and using an independent dataset (https://surfdrive.surf.nl/files/index.php/s/HTxdUbykBZm2cYM/download) provided by the BIDScoin^5^ team. Furthermore, the GitHub repository (https://github.com/bidsconvertr/bidsconvertr) and documentation (https://bidsconvertr.github.io/) facilitate user interaction in case of errors or questions.

### Description of the workflow

**Fig. 1:**
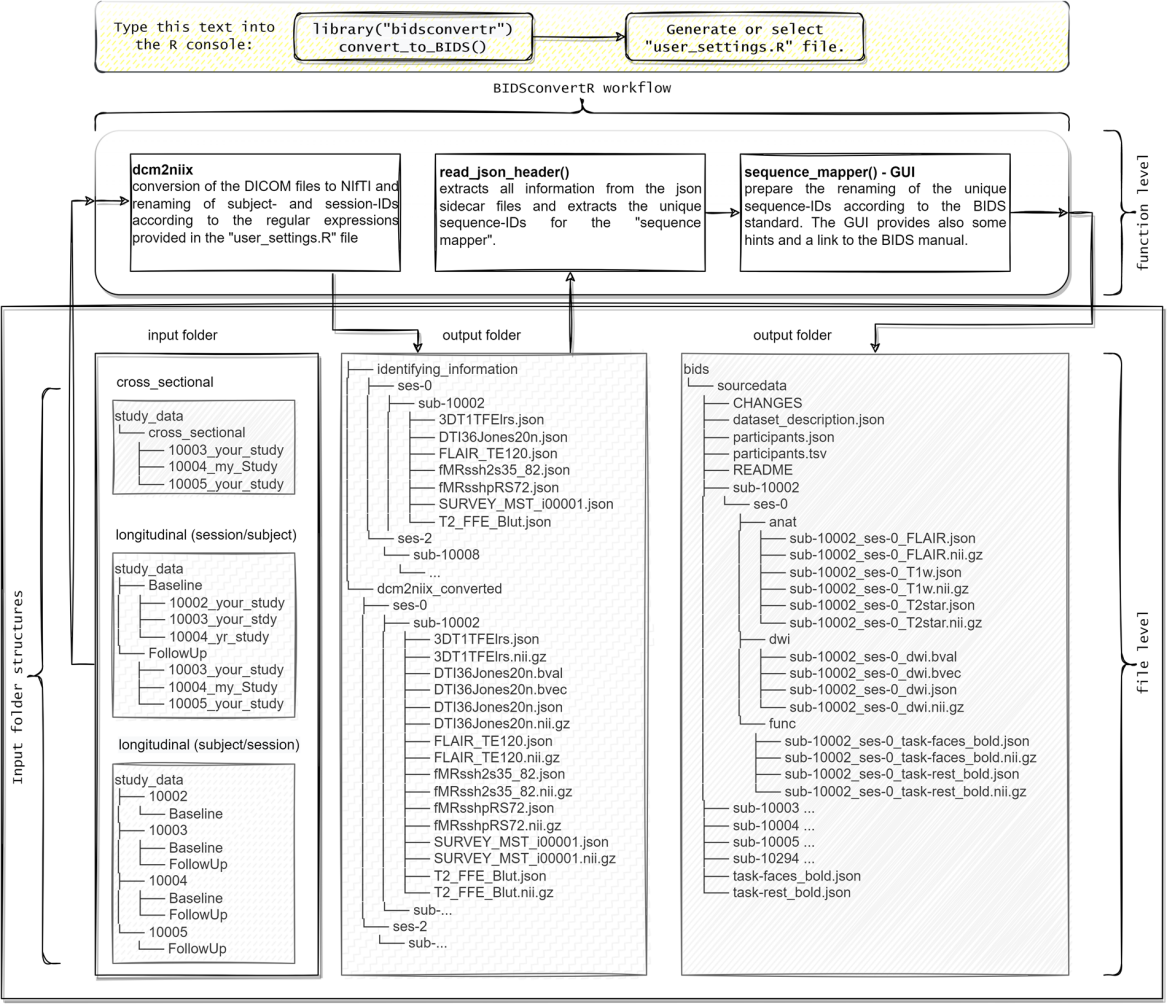
Visualization of an example data flow and the ‘convert_to_BIDS()’ function The input DICOM data will be indexed by subject-ID and session-ID. If the provided regular expressions match the data, all DICOM files will be converted and renamed according to BIDS to NIfTI. In the next step, all unique sequence-ID’s will be extracted and provided in an additional file. Now, the user has to use the “sequence mapper” graphical user interface to change these sequence-IDs to BIDS output sequence names and types and choose whether or not they should be stored to BIDS.

The BIDSconvertR’s workflow (Fig. 1) is condensed into a single function: convert_to_BIDS(). We provide a brief explanation of each step of this function below. The online documentation contains detailed information and screenshots of the workflow. Moreover, an online tutorial comprises a step-by-step walkthrough based on a sample dataset. The installation process is described in the usage notes below.

#### Getting started

To start the BIDSconvertR the package must be loaded (library(“bidsconvertr”)) and the (convert_to_BIDS()) function needs to be executed. Users can choose from existing “user_settings.R” files or create a new file from the file-selection and point-and-click pop-up boxes, which store information about file paths and input data structures. Each user interaction results in diagnostic output.

If users want to create a new file, a pop-up box appears with a user dialogue, where the user can select input and output paths with the file explorer, and is asked about the hierarchy of input folders to extract ‘session-’ and ‘subject-ID’. The required information is (1) the input path containing all subfolders with DICOMs, (2) a selection of the input folder hierarchy (choose between: session/subject or subject/session) and (3) the output folder for all temporary and BIDS files. At the end of the procedure the “user_settings.R” file is saved to the selected output folder.

The following step should enable the conversion in case of complicated subject-ID folder names into short and simple ones. The existing subject-IDs can optionally be shortened with regular expressions in the case of redundant prefixes, suffices, and strings. For instance, the template “[:digit:]{5}” means ‘extract any string in a filename with 5 digits’, and remove the rest of the string. Regardless of the setting, if no regular expression is added, the subject-IDs remain unchanged. Users then have the option to rename their session-IDs. Unchanged session-IDs are kept unchanged. The workflow creates background variables and file paths based on the “user_settings.R” file. If an already existing “user_settings.R” file is selected the pop-up-based user dialogue boxes are skipped.

#### From DICOM to NIfTI

A specified version of dcm2niix^4^ that worked best on our data will automatically be installed, although it is possible to install a custom version.

All DICOMs are converted twice with ‘dcm2niix’^4^ and then saved separately in a “dcm2niix_converted” and an “identifying_information” folder. The “dcm2niix_converted” folder contains NIfTI and JSON data with sensitive information removed from both files. The “identifying_information” folder contains only JSON sidecar files containing the sensitive privacy information (subject-ID, birthdate, age, gender, weight, height, acquisition date). Per folder, all JSON sidecar files are indexed, then the headers and all sequence parameters are extracted, merged together, and provided in one file (“json_metadata.tsv”) for quality control. Also, a file called “sequence_overview.tsv” is made with a summary of each sequence, including the amount of data for each sequence, in total and per session. This separation allows users to internally use personally identifiable information such as subject-IDs in pseudonymized datasets, while in the case of data sharing the metadata are de facto anonymized. However, additional removal of the facial structure and skull and removal of subject-IDs from filenames are still required for a fully anonymized dataset^10^.

#### From NIfTI to BIDS

**Fig. 2:**
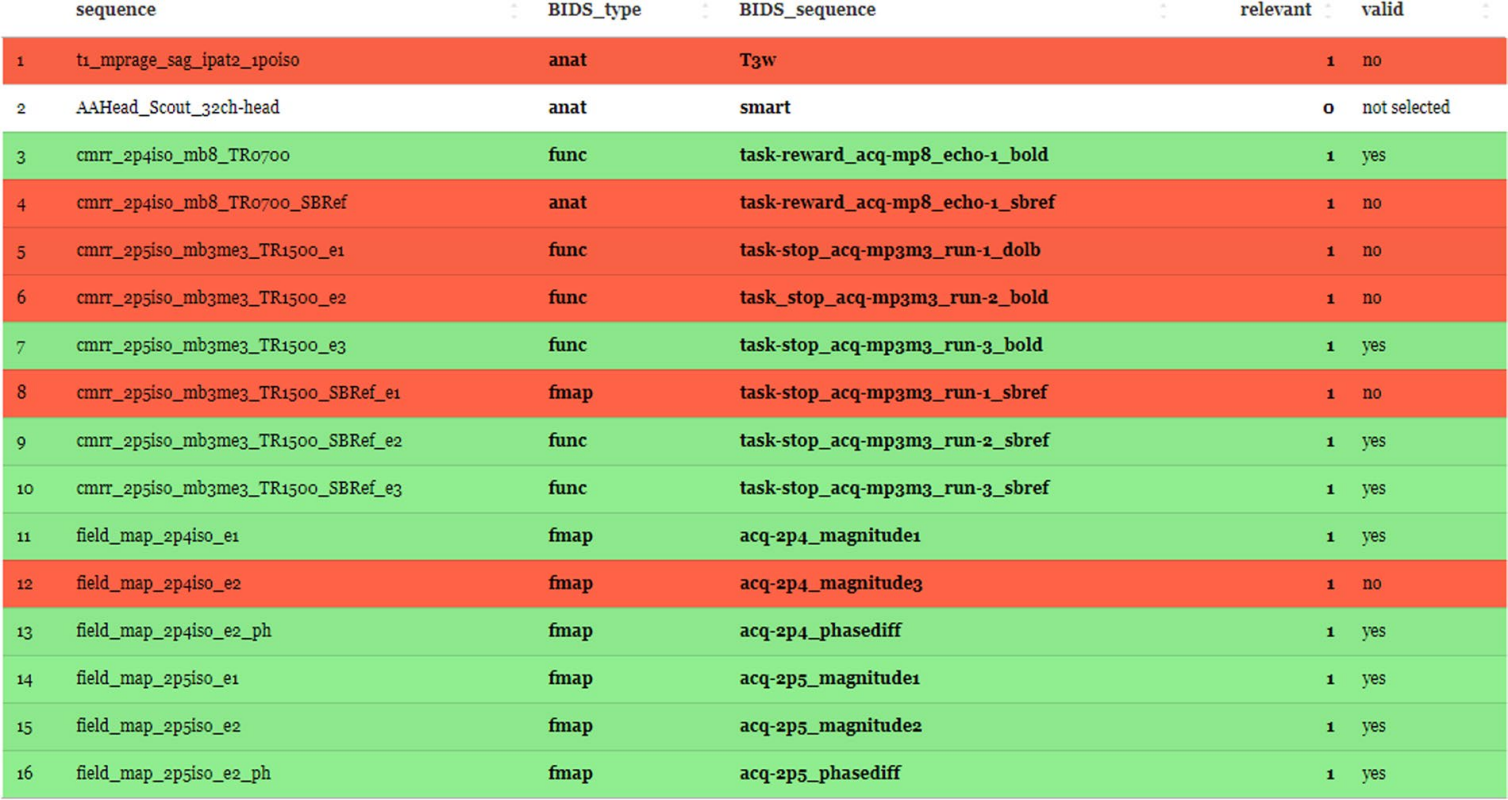
The ‘sequence mapper’ graphical user interface The interface starts automatically for the sequence mapping of the input sequence-IDs to BIDS sequence-IDs. The user has to edit all cells according to BIDS, click “save” and close the app. Please note the color-coding feature: Green rows indicate a valid BIDS sequence, while red rows incidate invalid BIDS sequences or cells that were not edited. Only the relevant marked sequences are copied into BIDS standard, while the white ones in the “relevant” column are skipped for conversion and validation.

The NIfTI filenames from the “dcm2niix_converted” folder must now be manually adapted to the BIDS specification. This is facilitated by the “sequence mapper” graphical user interface (GUI) (Fig. 2) that enables editing and live-validation of BIDS sequence IDs. The graphical user interface presents hyperlinks to the BIDS specification, which we recommend reading in its most recent version^2^ to ensure that the chosen names are in line with the most recent changes. The following information is required: (1) the BIDS sequence ID, (2) the BIDS type (“anat, dwi, func, fmap, or else”), and (3) the relevance (0 = no, 1 = copy to BIDS). The entries are color-coded in red and green, with green representing valid BIDS sequence names and red indicating invalid or unedited ones. The ‘sequence mapper’ (Fig. 2) does not allow workflow progress if the field is not edited or you enter an invalid BIDS type, BIDS sequence ID, or combination of both. When the GUI is closed and each field is edited, the workflow automatically starts with the subsequent step.

The NIfTI data are copied and renamed to BIDS specification^2^ inside the “bids” folder using the renaming pattern from the “sequence mapper” (Fig. 2). Each new file will be copied and renamed to the “bids” directory within the output folder. For each subject-ID there is an individual subfolder and within each of these subject-ID folders, the corresponding session-ID folders containing the sequence-type folders are created. The sequences can be found as NIfTI files at the lowest folder level (within the sequence-type folder). Each filename within the “bids” folder is specified as “subject-id_session-id_sequence-type_sequence-id” according to the BIDS naming convention (Fig. 1, right panel).

From here, all necessary BIDS metadata files are automatically generated. The “CHANGES”, “README” and “dataset_description.json” files contain placeholder content of the ‘BIDSconvertR’ package^6^, which needs to be updated by the users. It must be modified and adapted to their own data set. The sequence-agnostic BIDS JSON sidecar files (e.g., “participants.tsv”) are created automatically and contain the information from the anonymized JSON sidecar files in the “bids” folder. The metadata inside of the BIDS folder do not contain any potentially identifying information. Instead, the BIDSconvertR creates another “participants.tsv” in the “identyfing_information” folder that contains the privacy-sensitive information from the JSON headers.

### Diagnostics and quality control

**Fig. 3:**
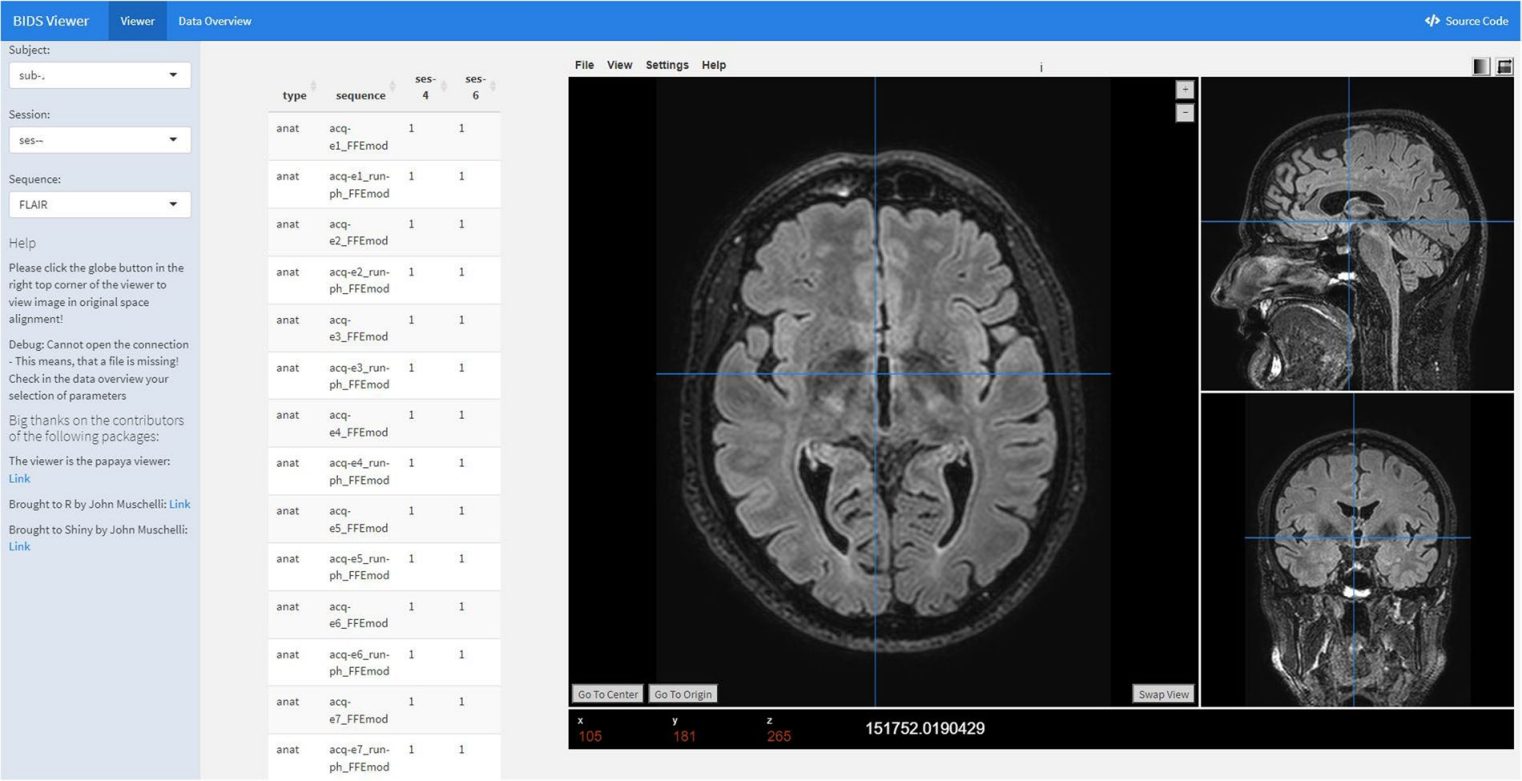
The ‘BIDS viewer’ graphical user interface The ‘BIDS viewer’ launches automatically from the ‘convert_to_BIDS()’ command when all files are converted to NIfTI and stored in the BIDS specification. The ‘papayaWidget’ viewer is used to check the visual quality control of the images. The user is able to inspect BIDS data easily without clicking through a nested folder structure. The viewer can also be used on existing BIDS datasets by giving the path as an argument to the ‘run_shiny_BIDS(“C:/path/to/BIDS_dataset/”)’ function.

The ‘BIDSconvertR’ features open-source dependencies: the recent version of the BIDS-validator^7^ and an embedded interactive MR image viewer based on papaya (https://CRAN.R-project.org/package=papayar, https://neuroconductor.org/package/papayaWidget) for visual quality control (Fig. 3). The BIDS-validator^7^ should be used to investigate potential issues with the resulting BIDS-dataset. If Docker is installed, the workflow automatically launches the latest Docker version of the BIDS-Validator in the “bids” folder. Otherwise, the corresponding website is launched. The final step is the automated start of the ‘Shiny BIDS’ viewer (Fig. 3). This GUI features an easy selection and navigation of the nested BIDS structure. It enables a manual inspection of the acquired data for quality control purposes with an image viewer based on papaya inside a graphical user interface. It can also be launched separately by using the run_shiny_BIDS() function and selecting a valid BIDS folder. After validation and visual quality control, users are asked to remove the “dcm2niix_converted” files, which are a temporary by-product of the workflow.

## Usage Notes

The following section describes the installation of the BIDSconvertR, gives some practical support for the use of regular expressions and ends with a tutorial which covers each step in the workflow of the “BIDSconvertR”^6^.

### Installation processes

Users need to install a recent version of R (https://www.R-project.org/, tested with 4.3.1) and RStudio (https://www.rstudio.com/). For a Windows system, ‘rtools43’ must additionally be downloaded and installed. When using a Debian (Ubuntu) system, dependencies must be installed with the following command: sudo apt install libcurl4-openssl-dev libssl-dev libxml2-dev libfontconfig.1-dev. For the installation of the BIDSconvertR the following two commands need to be entered on the R console inside RStudio: The ‘devtools’ package (https://CRAN.R-project.org/package=devtools) must be installed using the command install.packages(“devtools”). To install the ‘BIDSconvertR’ from GitHub, enter devtools::install_github(“bidsconvertr/bidsconvertr”). The package checks and installs all required libraries^11^ automatically during the installation process and asks for updates, if necessary.

### Optional feature: regular expressions

The use of regular expressions is an optional feature for the purpose of file cleaning. The following notes will deliver some basic knowledge of regular expressions by using some examples. Further information can be found in the “stringr” R package^12^ publication and cheat sheet, as well as in our BIDSconvertR documentation (https://bidsconvertr.github.io/usage_notes/#resources-for-regular-expressions).

Users can be very explicit regarding the regular expressions of the subject ID. If the subject ID consists of 5 digits, the regular expression is “[:digit:]{5}”. If the subject-ID consists of either “control_” or “intervention_” and two digits, the regular expression could look like this “(control_|intervention_)[:digit:]{2}”. The pipe operator “|” is used as a Boolean “OR” operator and can combine multiple regular expressions. Alternatively, the same expression can be generated by: “control_[:digit:]{2}|intervention_[:digit:]{2}”. It is recommended to use short and simple filenames. Since most of the acquisition data (e.g., date, sex) is included in the json sidecar header, it is not needed in the filename. One short and simple naming convention is one or two letters or digits defining the cohort, and sufficient digits corresponding to the intended study size (e.g., the first in a control cohort of size 100 could be named “c001”, or “i0001” from an intervention cohort of size 1000).

If there are dispensable letters, signs, or digits in the filename, they should be added to the “pattern to remove” expression. For instance, users can remove the strings “study”, “control” and “patient” with the regular expression: “(study|control|patient)”. To check for invalid subject-IDs, users can review the extracted subject-IDs in the diagnostic output in the terminal and the “dicom_paths.tsv” file. All non-matching strings of the filename are displayed in the “rest” columns of the “dicom_paths.tsv” file.

### Tutorial

The idea originates from the ‘BIDScoin’^5^ publication and is adapted to the ‘BIDSconvertR’. It guides users through the basic steps of the workflow on the basis of sample data. A more detailed (short) tutorial (https://bidsconvertr.github.io/tutorial/) and even more detailed usage notes (https://bidsconvertr.github.io/) that cover each step of BIDSconvertR can be found in the online documentation. Additionally, the tutorial dataset was used for validation of the ‘BIDSconvertR’ on an independent dataset.

#### Data preparation

Users can download the BIDScoin example data^5^ (https://surfdrive.surf.nl/files/index.php/s/HTxdUbykBZm2cYM/download). The data is compressed twice with gunzip (suffix: ‘.gz’) and tar (suffix: ‘.tar’) and needs to be unpacked. Users should then create a new folder (e.g. ‘bidscoin_example’) and copy the ‘raw’ subject folders into it.

#### The BIDSconvertR workflow

This are the main steps to use the ‘BIDSconvertR’ with the tutorial data:

1. Launch RStudio and use the R console from here on.
2. Load the library with the command: library(“bidsconvertr”).
3. Use the convert_to_BIDS() command to launch the tool.
4. Create your own ‘user_settings.R’ file by answering the questions in the text prompts.
  - Select the input folder that contains the DICOMs (e.g., ‘bidscoin_example_raw’).
  - Select the “../subject/session/..” order of folders.
  - Select the output folder.
  - Do not use the “subject-ID” and “pattern to remove” features (subject-IDs are valid).
5. Use the sequence mapper to rename your files according to BIDS, then save and close the app. See the online tutorial (https://bidsconvertr.github.io/tutorial/#default-values-for-tutorial-data) for a detailed map of the sequence names.
6. Now the data is saved in BIDS. The BIDS-Validator and the ‘Shiny BIDS viewer’ start.

## Discussion

The ‘BIDSconvertR’^6^ is a free, open-source R package and the first alternative for the R community. Most BIDS converters are developed in other languages such as Python or MATLAB (https://bids.neuroimaging.io/benefits.html). The ‘BIDSconvertR’ extends the capabilities of BIDS^2^ to users with a basic understanding of R and allows them to develop new features for the tool. It is simple enough that no programming experience is required to follow the tutorial (https://bidsconvertr.github.io/tutorial/) and use the package. In addition, the GitHub repository (https://github.com/bidsconvertr/bidsconvertr) and documentation (https://bidsconvertr.github.io/) facilitate user interaction in case of errors or questions.

To our knowledge, it is one of the few tools (BIDScoin^5^, Horos (https://horosproject.org/faqs/), Osirix^13^, and BrkRaw^14^) (https://bids.neuroimaging.io/benefits.html) with a graphical user interface. In contrast to the others it simplifies the ‘sequence mapping’ with a live color-coded validation of the entered sequences and features a viewer for visual inspection of BIDS-structured MRI images.

Our tool is developed to:

- be executed continuously during the data acquisition of a study, as it checks for already converted and existing data,
- facilitate the creation of short and simple filenames,
- check and validate ongoing changes in sequence names and types,
- extract and merge all metadata information,
- detect implausibilities in sequence parameters by color-coding,
- create automatically required sequence-agnostic files like “participants.tsv”,
- validate the dataset with BIDS-Validator^7^ in Docker or the website, and
- efficiently inspect and navigate through BIDS structured MRI data with the ‘Shiny BIDS viewer’.

The ‘BIDSconvertR’ has the some limitations. It was developed only with MRI data and up to now supports only this type of data. The program requires an IDE (integrated development environment, here RStudio) and a console, which is however also a requirement for all other BIDS converters. To facilitate users that are unfamiliar with R the use of the ‘BIDSconvertR’ we linked several resources in this publication and the online documentation (https://bidsconvertr.github.io). A basic understanding of regular expressions is required for advanced features, like the cleaning of subject IDs. Although regular expressions are complicated to use, the documentation of the ‘BIDSconvertR’ should help the user in the application and describe the installation, workflow, and its practical use with a tutorial. Moreover, the use of regular expressions is optional and not required for the basic use of the ‘BIDSconvertR’.

Taken together, the BIDSconvertR is the first BIDS converter developed in R, which is aimed at users with a background in R (statistics or epidemiology) to get their data into BIDS without the necessity to learn Python or MATLAB and their ecosystem. In a future update, we plan to extend the ‘BIDSconvertR’^6^ with additional wrapper functions for popular processing pipelines like fMRIPrep^15^ or MRIQC^16^, allowing users to process their MRI data even more efficiently.

## Methods

### Study data

The data used for the development of the tool originate from the BiDirect study and is briefly described here. The BiDirect study^8,9^ is a prospective cohort study aiming to establish the bidirectional relationship between subclinical arteriosclerosis and depression. Data were acquired over a period of 12 years, including repeated MR-imaging of the brain using the same Philips scanner and sequences. The study comprised approximately 90,000 images from around 4,500 MRI sessions. The ‘BIDSconvertR’ uses ‘dcm2niix’ to convert DICOM data. Data from all major MRI vendors supported by ‘dcm2niix’ can be converted. Conversion was tested on three data sets: The BiDirect MRI data that were acquired with a Philips scanner, and two data sets from Siemens scanners, one in-house dataset and the example data provided by the BIDScoin developers^5^. A recent version of ‘dcm2niix’ (V1.0.20230411, Chris Rorden^4^) was implemented and tested for compatibility.

### Software & hardware

The ‘BIDSconvertR’ package^6^ was developed with R 4.3.1 and RStudio. The packages ‘usethis’(https://CRAN.R-project.org/package=usethis) and ‘devtools’ (https://CRAN.R-project.org/package=devtools) were used for package development. The ‘tidyverse’^11^ functions of the packages ‘dplyr’, ‘tidyr’, ‘stringr’ were used for file renaming and restructuring. JSON files were extracted with the package ‘rjson’ (https://CRAN.R-project.org/package=rjson). The ‘Sequence Mapper’ GUI uses the ‘shiny’ (https://CRAN.R-project.org/package=shiny) and ‘DT’ (https://CRAN.R-project.org/package=DT) packages for the spreadsheet interaction and color-coded BIDS validation. The ‘Shiny BIDS viewer’ was made with ‘flexdashboard’ (https://CRAN.R-project.org/package=flexdashboard), ‘shiny’, and papaya. The conversion from DICOM to NIfTI is done by ‘dcm2niix’ (V1.0.20211006, Chris Rorden^4^), from which each feature can be implemented in the workflow. All code is hosted freely and open-source on a GitHub repository^6^.

### Requirements

In general, there are a few requirements (for the data, user, and system) for the ‘BIDSconvertR’^6^: Users needs a basic understanding of the BIDS nomenclature and file management. In the case of advanced features like file cleaning, a basic understanding of regular expressions is required. Users can find useful resources on our Github README (https://github.com/bidsconvertr/bidsconvertr) or in the documentation (https://bidsconvertr.github.io/).

### Input file convention

Data must be in a specific structure that corresponds to the common output of PACS (Picture Archiving and Communication System): either (1) a folder per time point or study, containing subfolders of each subject with the DICOM data (“input/session/subject”) or (2) a folder per subject, containing the timepoint subfolders with the DICOM data (“input/subject/session”) (Fig. 1, left side). If there are only cross-sectional data, we recommend to create a folder named, e.g. “cross_sectional”, “baseline” or “0”, that contains all subject folders with the DICOM data. If data are structured another way, we recommend to rearrange it. To set up the hierarchical order in which the ‘BIDSconvertR’ extracts information from the data, users are required to set the option during the user dialogue either to ‘session_subject’ or ‘subject_session’. In general, we recommend removing spaces or special text symbols in file or folder names. If the data already have the BIDS-required prefixes “sub-” or “ses-”, users do not need to change it.

## Data Availability

Each release is saved at Zenodo^6^, which can also be accessed via 10.5281/zenodo.5878407. We thank the developers of BIDScoin^5^ for the permission to use the sample data in the tutorial, which can be downloaded at https://surfdrive.surf.nl/files/index.php/s/HTxdUbykBZm2cYM/download.

## Code availability

All code is hosted freely and open-source on a GitHub repository, accessible via https://github.com/bidsconvertr/bidsconvertr. The documentation is hosted on the GitHub page: https://bidsconvertr.github.io.
